# FABP5 Inhibition against *PTEN*-Mutant Therapy Resistant Prostate Cancer

**DOI:** 10.3390/cancers16010060

**Published:** 2023-12-21

**Authors:** Manojit M. Swamynathan, Grinu Mathew, Andrei Aziz, Chris Gordon, Andrew Hillowe, Hehe Wang, Aashna Jhaveri, Jude Kendall, Hilary Cox, Michael Giarrizzo, Gissou Azabdaftari, Robert C. Rizzo, Sarah D. Diermeier, Iwao Ojima, Agnieszka B. Bialkowska, Martin Kaczocha, Lloyd C. Trotman

**Affiliations:** 1 Cold Spring Harbor Laboratory, Cold Spring Harbor, NY 11724, USAaashnajhaveri01@gmail.com (A.J.);; 2 Department of Molecular and Cell Biology, Stony Brook University, Stony Brook, NY 11794, USA; 3 The Eppley Institute for Research in Cancer and Allied Diseases, Fred & Pamela Buffett Cancer Center, University of Nebraska Medical Center, Omaha, NE 68198, USA; 4 Department of Anesthesiology, Stony Brook University, Stony Brook, NY 11794, USA; chris.gordon2@stonybrookmedicine.edu (C.G.); andrew.hillowe@stonybrook.edu (A.H.);; 5 Department of Chemistry, Stony Brook University, Stony Brook, NY 11794, USAiwao.ojima@stonybrook.edu (I.O.); 6 Department of Medicine, Stony Brook University, Stony Brook, NY 11794, USA; michael.giarrizzo@stonybrookmedicine.edu (M.G.); agnieszka.bialkowska@stonybrookmedicine.edu (A.B.B.); 7 Department of Anatomic Pathology, Stony Brook University, Stony Brook, NY 11794, USA; 8 Department of Applied Mathematics and Statistics, Stony Brook University, Stony Brook, NY 11794, USA; 9 Institute of Chemical Biology and Drug Discovery, Stony Brook University, Stony Brook, NY 11794, USA; 10 Department of Biochemistry, University of Otago, Dunedin 9016, New Zealand; sarah.diermeier@otago.ac.nz

**Keywords:** prostate cancer (PC), PTEN tumor suppressor, lipid signaling, fatty acid binding proteins (FABPs), FABP5, SBFI-103, enzalutamide resistance, apalutamide resistance, taxane resistance, genetically engineered mouse (GEM) models of human cancer, RapidCaP, castration-resistant prostate cancer (CRPC), metastatic castration-resistant prostate cancer (mCRPC), androgen deprivation therapy (ADT)

## Abstract

**Simple Summary:**

Prostate cancer therapy suffers from a lack of effective targets because, typically, success with blockade of the androgen receptor gives way to drug resistance and lethal disease relapse. Large scale genome sequencing efforts have demonstrated that lethal recurrent disease most often presents with loss of the *PTEN* and *TP53* tumor suppressors. Unfortunately, the systematic testing of PTEN/ PI 3-Kinase pathway-specific inhibitors has shown only limited results in prostate cancer trials. Thus, there are currently no FDA-approved drugs targeting this axis in prostate cancer patients. Here we propose a new target, the FABP5 lipid carrier. *FABP5* amplification and surge in expression are strongly correlated to that of the *MYC* oncogene, a known driver of advanced *PTEN*-deficient prostate cancer. Here, we present a new pre-clinical platform to assess the efficacy and biology of inhibiting FABP5 with small molecules. Our platform is based on a PTEN-deficient prostate cancer cell type that is insensitive to standard of care therapies.

**Abstract:**

Resistance to standard of care taxane and androgen deprivation therapy (ADT) causes the vast majority of prostate cancer (PC) deaths worldwide. We have developed RapidCaP, an autochthonous genetically engineered mouse model of PC. It is driven by the loss of PTEN and p53, the most common driver events in PC patients with life-threatening diseases. As in human ADT, surgical castration of RapidCaP animals invariably results in disease relapse and death from the metastatic disease burden. Fatty Acid Binding Proteins (FABPs) are a large family of signaling lipid carriers. They have been suggested as drivers of multiple cancer types. Here we combine analysis of primary cancer cells from RapidCaP (RCaP cells) with large-scale patient datasets to show that among the 10 FABP paralogs, FABP5 is the PC-relevant target. Next, we show that RCaP cells are uniquely insensitive to both ADT and taxane treatment compared to a panel of human PC cell lines. Yet, they share an exquisite sensitivity to the small-molecule FABP5 inhibitor SBFI-103. We show that SBFI-103 is well tolerated and can strongly eliminate RCaP tumor cells in vivo. This provides a pre-clinical platform to fight incurable PC and suggests an important role for FABP5 in *PTEN*-deficient PC.

## 1. Introduction

Prostate cancer (PC) remains the second-leading cause of male cancer deaths in the U.S., with one in eight men developing invasive carcinoma over a lifetime [1]. Landmark bulk tumor analyses [2,3,4], as well as single cell resolution studies [5], point to a single cell of origin, expanding through truncal evolution to periodically overcome the major bottlenecks of metastasis and anti-hormonal therapy. As shown by contrasting primary with metastatic disease [6], we now know that spontaneous deletions of *PTEN* and *TP53* are uniquely and selectively enriched in human metastatic PC, together with amplification of the androgen receptor (AR). These analyses collectively suggest that *PTEN*/*TP53* loss both precedes and can drive metastasis, a hypothesis that is functionally validated in multiple Genetically Engineered Mouse (GEM) models of PC [7]. In contrast, AR amplification is typically triggered by androgen deprivation therapy (ADT) and results in metastatic castration-resistant PC (mCRPC) [8,9]. Although localized PC is treatable if detected early, mCRPC typically becomes incurable even after taking innovative approaches with the latest generations of drugs that directly target the androgen receptor axis of the cancer cell [10]. This *status quo* now highlights two specific needs: (1) the need for new drugs against new targets that effectively kill those cells that have acquired the metastatic and therapy-resistant properties, and (2) cell types that faithfully represent these resistance properties so that new drugs and targets can be tested rigorously.

Fatty acid binding proteins (FABPs) are intracellular proteins that transport lipids to nuclear receptors, whose activation promotes tumor growth and metastasis [11,12,13]. Preclinical and clinical evidence indicates that FABP5, while not expressed in the normal prostate, becomes highly expressed in advanced metastatic prostate cancer and that its expression is linked with reduced patient survival [14,15,16]. Aggressive PC is characterized by dramatically elevated fatty acid metabolism and signaling [17,18,19]. Previous work by us and others suggested that FABP5 functions as a major node in a prostate cancer lipid signaling network by linking cytoplasmic lipid production to nuclear receptor signaling [20,21,22]. From this, it followed that FABP5 could be a novel target for the treatment of mCRPC [23].

Genetically engineered mouse (GEM) models are the gold standard for modeling human cancer because disease progression can be studied in a fully native setting in a much faster time frame. Specifically, GEM models based on somatic gene transfer into the target tissue have been developed and used with great success for understanding the mechanisms behind lethal cancer progression in lung and brain tumors [24,25,26,27]. We use a somatic gene transfer GEM model for advanced prostate cancer termed RapidCaP [28]. This system allows us to go beyond the study of PC progression by revealing mechanisms of endogenous metastasis, e.g., bone, lung, and liver [29]. When testing the efficacy of anti-androgen therapy in RapidCaP, we found that in the short term (weeks), metastatic disease can respond to castration, albeit variably, similar to what is observed in patients. However, in the long term (months), RapidCaP mice invariably present with lethal disease relapse after castration. Thus, this model presents us with an experimental platform to uncover and validate new therapy approaches that can outperform the standard of care.

## 2. Methods

### 2.1. Cell Lines and Culture

All cell lines were tested for mycoplasma in-house, and the human-derived cancer cell lines were authenticated by STR profiling at an external facility (Genetics core, University of Arizona). 22Rv1, DU145, LNCaP, PC3, and RCaP cells were cultured in RPMI-1640 supplemented with 10% fetal bovine serum (FBS) and penicillin–streptomycin. All cell lines were grown at 37 °C with 5% CO_2_.

### 2.2. Cell Viability

2500 cells per well were seeded in low serum media (2% FBS containing RPMI) on a 96-well plate and left to adhere overnight. The next day, the cells were treated with a dilution series of the small molecule in 2% FBS containing RPMI for 72 h. To measure cell viability, the media containing the compound was then removed and MTT solution was added. After labeling cells with MTT, the absorbance was read at 570 nM using the SpectraMax plate reader (Molecular Devices). Alternatively, cell viability was measured immediately post-drug treatment with Cell Titer Glo (G7573, Promega, Madison, WI, USA), per the manufacturer’s instructions, on a SpectraMax plate reader (Molecular Devices). Cell viability in test conditions is reported as a percentage relative to the vehicle-only treated condition. The data were graphically displayed using GraphPad Prism 9 (GraphPad Software, Boston, MA, USA). Each point (mean ± standard error of mean) represents the growth of treated cells compared to vehicle-only-treated cells. The curves were fitted using a non-linear regression model with a sigmoidal dose response.

### 2.3. Animal Trials

100,000 RcaP cells were mixed with an equal volume of matrigel and then subcutaneously injected into Nu/J mice (JAX stock #002019). The tumors were allowed to be engrafted, and post-establishment (2 weeks post-injection), mice were enrolled on a randomized basis to be treated with vehicle, α-SB-FI-103 (20 mg/kg), or α-SB-FI-103 (40 mg/kg). The treatment was delivered via intraperitoneal (i.p.) injections on a daily basis. Tumor volumes were measured using caliper measurements using the following formula: Tumor Volume [mm^3^] = (length [mm] × width [mm] × width [mm])/2.

For immunohistochemistry analysis, tumors from 12 trial animals (4 animals per trial arm) were processed as described using the Roche Discovery XT automated staining platform as described previously [28]. Animal work was carried out under IACUC/IRB-approved protocols for CSHL #23-20-17-14-11-08-3 and SB-SUNY #850980.

### 2.4. Histology Analysis and Quantification

For H&E staining, five-micrometer FFPE sections of tissues were baked in a 65 °C oven overnight and subsequently deparaffinized in xylene, rehydrated by incubation in a decreasing ethanol bath series (100%, 95%, and 70%), and stained with Hematoxylin Stain Solution, Gill 3 (Ricca Chemical Company (Arlington, TX, USA): 3537-32) and Eosin Y (Sigma–Aldrich (Saint Louis, MO, USA): HT110216). Sections were dehydrated in an increasing series of ethanol baths (70%, 95%, and 100%), cleared in xylene, and mounted with Cytoseal XYL xylene-based mounting media (Thermo Scientific (Waltham, MA, USA): 8312-4). The H&E stains were used for histopathological assessment. Images were taken using a Nikon Eclipse 90i microscope (Nikon, Melville, NY, USA) and processed with NIS-Elements (Nikon, Melville, NY, USA).

### 2.5. Immunofluorescence and Immunohistochemistry Staining

Five-micrometer FFPE sections of tissues were baked in a 65 °C oven overnight and subsequently deparaffinized in xylene, rehydrated by incubation in a decreasing ethanol bath series (100%, 95%, and 70%), followed by antigen retrieval in citrate buffer solution (10 mM sodium citrate, 0.05% Tween-20, pH 6.0) at 110 °C for 10 min using a decloaking chamber (Biocare Medical, Pacheco, CA, USA) and 30 min incubation at 4 °C. The histological sections were incubated with blocking buffer (5% bovine serum albumin and 0.01% Tween 20 in 1× Tris-buffered phosphate-buffered saline [TTBS]) for 1 h at 37 °C. The primary antibodies rabbit anti-RFP (1:300; Rockland Immunochemicals, Limerick, PA, USA: 600-401-379) and rat anti-MKI67 (1:300; DAKO: M7249) were added and tissues were incubated at 4 °C overnight. The following day, secondary Alexa Fluor-labeled secondary antibodies (1:300 goat anti-rabbit RRX and 1:800 goat anti-rat AF 647) were added in blocking buffer for 30 min at 37 °C, counterstained with Hoechst 33258 (ThermoFisher Scientific: H3569), mounted with Fluoromount Aqueous Mounting Medium (Sigma–Aldrich: F4680), and coverslipped. For immunohistochemistry, rabbit anti-RFP (1:300; Rockland: 600-401-379) and rabbit anti-MMP9 (1:1000; Abcam: ab283575) were added, and tissues were incubated at 4 °C overnight. MACH 3 rabbit HRP polymer detection (Biocare Medical, Cat. M3R531L) and Betazoid DAB (Biocare Medical, Cat. BDB2004L) were used for the detection. The slides were dehydrated, cleared in xylene, and mounted with Richard–Allan Scientific^®^ Cytoseal™ X.Y.L. Mounting Medium (Fisher Scientific (Waltham, MA, USA), 8312-4). Slides were analyzed using a Nikon eclipse 90i microscope (Nikon Instruments Inc.) equipped with DS-Qi1Mc and DS-Fi1 CCD cameras (Nikon Instruments Inc.). Images (4 different sections per tumor) were quantified using the Imaris 10.0 imaging software from Oxford Instruments (Bitplane AG, Zurich, Switzerland) for immunofluorescence staining and manually for chromogenic staining.

### 2.6. Western Blotting

Whole cell lysates were prepared by removing the culture media, washing with cold PBS, and lysing cells with 1x RIPA buffer containing Mini-complete protease inhibitors (11836170001, Roche, Basel, Switzerland) and phosphatase inhibitor cocktail (4906845001, Roche). Protein concentrations were determined using the Bradford assay, and 20 μg of protein/well was loaded onto the gels. Standard western blotting procedures were followed, and the following antibodies were used: β-ACTIN (Sigma–Aldrich, A3854), FABP5 (Cell Signaling Technology (Danvers, MA, USA), 39926), AR (Santa Cruz Biotechnology (Dallas, TX, USA), sc-7305), FKBP5 (Cell Signaling Technology, 12210S), NKX3.1 (Santa Cruz Biotechnology, sc-393190), β-Tubulin (Cell Signaling Technology, 2146), and Vinculin (Cell Signaling Technology, 13901). Western blots were imaged using Amersham Hyperfilm ECL (Amersham Bioscience, Buckinghamshire, UK), and the scanned images were analyzed using ImageJ software, v. 1.54f).

### 2.7. RNA Sequencing

RNA was extracted from cells stored in TRIzol, -80C (TRIzol Reagent, Thermo Fisher Scientific, cat# 15596026), following the Direct-Zol RNA MiniPrep instruction manual from ZymoResearch (Cat# R2050). The quantity and quality of RNA were determined by Nanodrop. 1μg RNA was converted to cDNA following the Reverse Transcription Kit instructions from Applied Biosystems (high-capacity cDNA Reverse Transcription Kit Cat# 4368813). RNA sequencing was performed using Illumina’s NovaSeq platform with unstranded library preparation. Single-end reads were generated with >19 million reads per sample and a mean quality score >35. The transcript data in fastq files were aligned to the genome using STAR (version 2.7.9a); the genome index was generated using the GRCm 38 primary assembly genome fasta. QC was conducted prior to and after alignment using FastQC and PicardTools, respectively. Transcript quantification was obtained using either STAR counts or Salmon (version 1.5.2). Differential analysis was conducted using DESeq2, and fragments per kilobase of transcript per million mapped reads (FPKM) were exported. RNA sequencing data generated in this study have been deposited in the Gene Expression Omnibus (http://www.ncbi.nlm.nih.gov/geo/) with the accession number GSM7951577.

### 2.8. Analysis of Publicly Available Datasets

Gene copy number alteration was explored and visualized using the cBIO platform (cbioportal.org) using this Onco Query Language (OQL) for refinement of gene status: Datatypes: MUT AMP HOMDEL HETLOSS GAIN. For RNA expression analysis, we selected the “mRNA expression z-scores relative to diploid samples (RNA Seq V2 RSEM)” option as a criteria with a z = score threshold of 1.5 standard deviations. Visualization and localization of chromosome 8 genes and their copy number status on a PC were done using Nexus Copy Number Software v. 10.0 (now Bionano: bionano.com/nexus-copy-number-software/). False discovery rates (q bounds) were determined as described in [30]. Genetic dependency and gene expression data for AR in cancer cell lines were downloaded from the Cancer Dependency Map (DepMap) portal version 21Q3/22Q4 [31]. The data were graphically displayed using GraphPad Prism 9.4.1.

### 2.9. Statistical Analysis

When appropriate, the analysis was performed using parametric and non-parametric statistical analysis, with a value of *p* < 0.05 considered significant. This analysis used GraphPad Prism version 9.4.1 for macOS (GraphPad Software, Boston, MA, USA).

## 3. Results

### 3.1. The FABP Family in Aggressive Prostate Cancer: Human and Mouse

**Figure 1:**
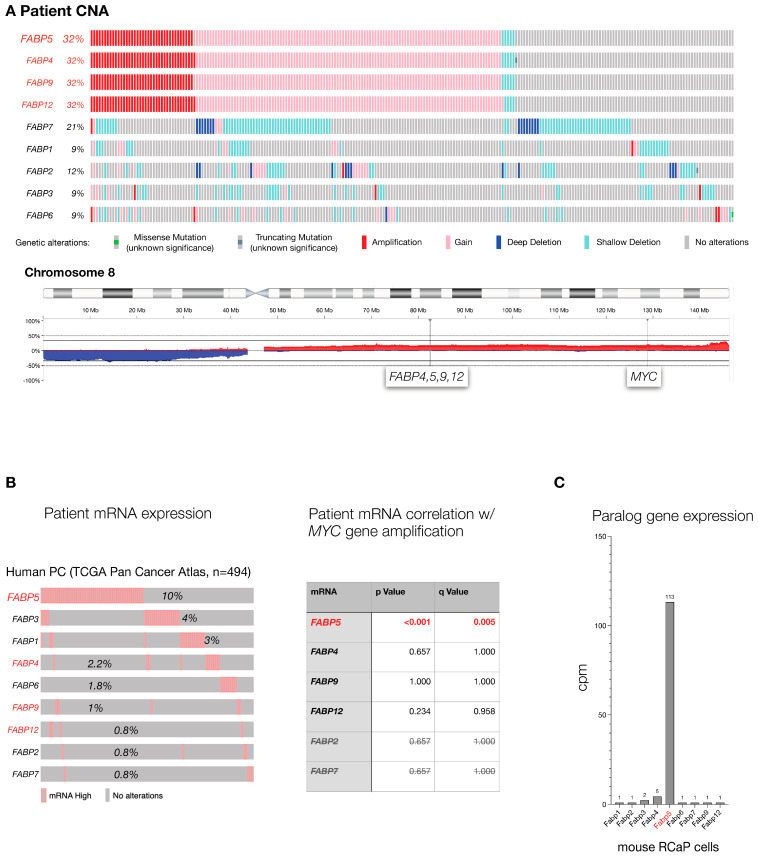
The *FABP* gene family in aggressive prostate cancer: (**A**), Top: Mutation frequency of *FABP* paralogs in TCGA cohort of primary prostate adenocarcinoma as curated at cBIOPortal (TCGA, PanCancer Atlas, 494 total samples). Bottom: Genomic location of *FABP4,5,9,12* and distance to *MYC* gene on human chromosome 8. (**B**), Left panel: analysis of *FABP* paralog mRNA expression levels in the TCGA cohort shown in (**A**). Paralogs that are neighbors and co-amplified on chromosome 8 (**A**) are highlighted in red. Right panel: significance of correlation between *FABP* mRNA expression and *MYC* gene amplification in TCGA patient cohort. *p* values were calculated using Student’s *t*-test, and q-bound shows false discovery rate (see Section 2). (**C**), expression levels of the *Fabp* paralogs in RCaP cells as determined by RNA sequencing.

The *FABP* genes are a family of 10 genetic paralogs that function in lipid signaling [32,33]. To test which of the *FABP* genes are most altered in human PC, we analyzed the TCGA prostate cancer genome data set (TCGA, Firehouse Legacy) curated at cBIOPortal [34,35] at cbioportal.org. This data set contains 489 primary PC samples that were comprehensively profiled for genome-wide copy number alteration, the driving force of prostate cancer, gene mutations, and gene expression changes that compare normal prostate to tumor. As shown (Figure 1A), *FABPs 4, 5, 9,* and *12* gene copy numbers are altered in one-third of the analyzed tumors. This result is driven by a shared pattern of co-amplification due to the close proximity of the four paralogs’ genomic loci. Furthermore, the high amplification frequency in PC is due to their location on the long arm of chromosome (chr.) 8: the chr. 8q whole arm amplification is among the most frequent events of (prostate) cancer. To disentangle their genomic location from gene correlation, we next studied tumor-associated increases in mRNA expression as an individual gene filter that is orthogonal to gene amplification. As shown in Figure 1B (left), *FABP5* topped the cancer-specific high-expression list of *FABP* paralogs, especially among those located on chromosome 8q (shown in red). This indicated that among the chr.8q *FABPs*, only *FABP5* amplification actually results in higher mRNA, which is a prerequisite for any functional consequence of the gene amplification. Furthermore (see Figure 1B, right), we found that *FABP5* high mRNA expression is significantly correlated with *MYC* gene amplification, a well-validated driver gene of prostate cancer. In contrast, expression levels of the other *FABPs* were not correlated with amplification of *MYC*, even for the paralogs that also reside on chromosome 8q (see Figure 1B right, *FABP4*, *-9*, *-12*). Finally, we used our mouse model to study FABP family mRNA levels in prostate cancer cells derived from a RapidCaP tumor (RCaP cells, described below). As shown in Figure 1C, while most FABPs were barely detectable, only *Fabp5* mRNA was highly expressed. Taken together, our cross-species cancer genomics analysis points to *FABP5* as the PC relevant candidate driver of the gene family.

### 3.2. RapidCaP Derived Cells as Proxies for Incurable PC

**Figure 2:**
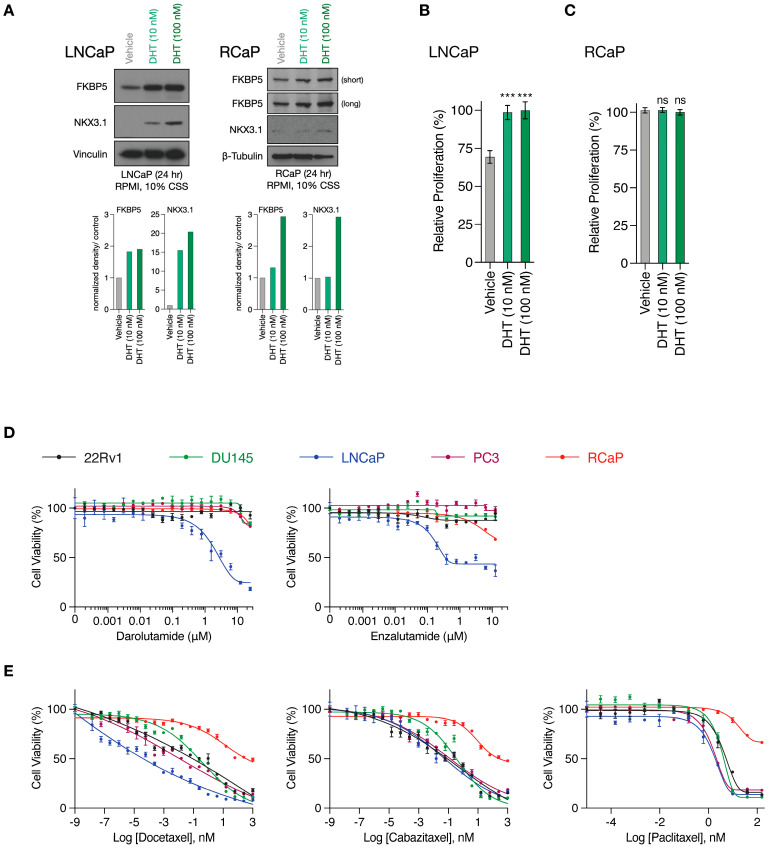
RapidCaP derived cells are resistant to standard of care anti-androgens and taxanes. (**A**), Top, Western blot analysis of AR target genes in whole cell lysates prepared from LNCaP (left) and RCaP (right) cells cultured in charcoal-stripped serum (CSS)-containing media following 24 h stimulation with DHT. Bottom: Quantification of bands normalized to the loading control and relative to vehicle treatment. (**B**), Relative proliferation of LNCaP cells cultured in charcoal-stripped serum (CSS)-containing media in the presence or absence of DHT for 96 h. (**C**), Relative proliferation of RCaP cells cultured in charcoal-stripped serum (CSS)-containing media in the presence or absence of DHT for 96 h. Data are mean ± s.d. *p* values were calculated using one-way ANOVA with Tukey’s post hoc test. ns, not significant. *** *p* < 0.001. (**D**), Cell viability curves of the indicated prostate cancer cell lines treated with increasing concentrations of AR antagonists (Darolutamide and Enzalutamide) for 72 h (*n* = 3 biologically independent samples). Data are mean ± s.d. (**E**), cell viability curves of the indicated prostate cancer cell lines treated with increasing concentrations of microtubule inhibitors (Docetaxel, Cabazitaxel, and Paclitaxel) for 72 h (*n* = 3 biologically independent samples). Data are mean ± s.d. The original western blots of Figure 2A are shown in Figure S4.

RapidCaP tumorigenesis is initiated through somatic gene transfer of Cre-recombinase by virus injection directly into the prostates of *Pten*^loxP/loxP^; *Trp53*^loxP/loxP^ mice. This results in disease initiation in only a few cells [28], which expand to form lesions involving thousands of cells within weeks, as revealed by the inclusion of a fluorescent protein marker (lox-stop-lox tdTomatoFP) in the mouse germ line, combined with 3D-organ imaging of the prostate [36]. To more seamlessly transition between in vitro and in vivo pre-clinical modeling, we have isolated primary PC cells from Td-Tomato-positive RapidCaP lesions (RCaP cells, Supplementary Figure S1). As shown above (Figure 1C), these cells expressed high levels of *Fabp5*. They also expressed the androgen receptor (*Ar*) together with its target gene *Fkbp5,* and we saw that addition of the AR ligand dihydrotestosterone (DHT) to culture media led to nuclear translocation of mouse AR (Supplementary Figure S2A, Supplementary Figure S4). Next, we tested if there is a functional AR response by analyzing the NKX3.1 and FKBP5 target proteins using the androgen-sensitive and -dependent LNCaP PC cell line as a reference (Supplementary Figure S2B). Compared to LNCaP cells, the RCaP cells showed a weak NKX3.1 response (Figure 2A, Supplementary Figure S4), and importantly, they did not respond to DHT supplementation (Figure 2B,C). This suggests that any residual AR function is uncoupled from their proliferation. Next, we studied the effects of the two clinically relevant AR antagonists, darolutamide [37] and enzalutamide [38], which are known to inhibit AR function through several mechanisms: competitive inhibition of the DHT ligand, suppression of nuclear translocation, interference with DNA binding, and co-activator recruitment [39]. We tested effects in RCaP versus a panel of four human cell lines, including LNCaP, PC3, and DU145, which come from metastatic sites (lymph node, bone, and brain, respectively), and the 22Rv1 PC cell line, which is derived from a localized prostate tumor (see Supplementary Figure S2C for cancer genome profiling). We performed exponential dose escalation curves and measured cell viability. This showed that the LNCaP cells were sensitive to both drugs in the nanomolar range (Figure 2D). In contrast, however, the RCaP and other human cells were largely resistant to both drugs beyond 1 µM. These results confirmed that the RCaP cells attained insensitivity to castration and thus presented a hallmark feature of the RapidCaP PC model [28]. Next, we tested RCaP sensitivity to taxanes, which are a standard of care PC chemotherapy that is used against castrate-resistant prostate cancer (CRPC). As shown in Figure 2E, RCaP cells showed a very poor response to docetaxel, cabazitaxel, and paclitaxel, which indeed set them apart from the tested human cell lines. Collectively, these data establish that the RCaP cells can be used as a murine-derived proxy cell type for incurable PC.

**Figure 3:**
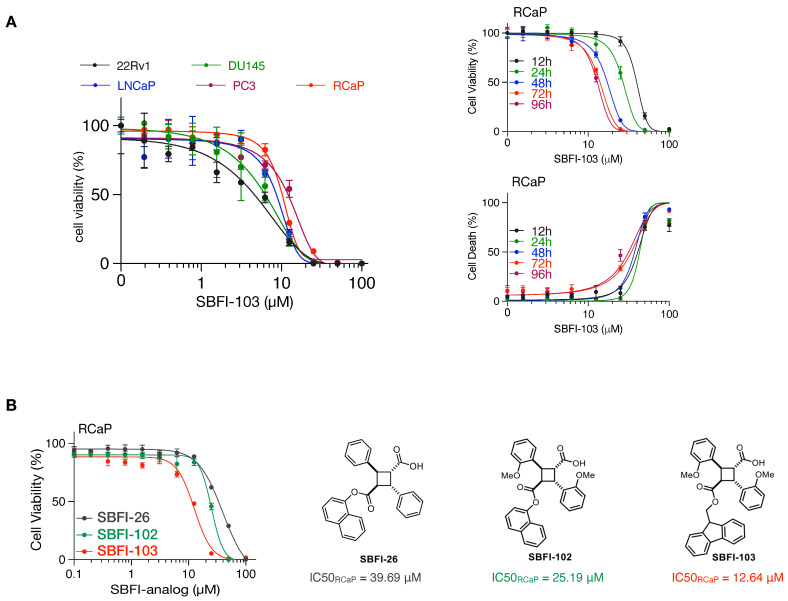
(**A**), Left: Cell viability curves of the indicated prostate cancer cell lines treated with increasing concentrations of SBFI-103 for 72 h (*n* = 3 biologically independent samples). Data are mean ± s.d. Right-top: cell viability curves of RCaP cells treated with increasing concentrations of SBFI-103 for the indicated time points (*n* = 4 biologically independent samples). Right-bottom: percent cell death indicated by propidium iodide uptake of RCaP cells treated with increasing concentrations of SBFI-103 (*n* = 4 biologically independent samples). Data are mean ± s.d. (**B**). Left, cell viability curves of RCaP cells treated with increasing concentrations of 3 TAME inhibitors of FABP5 for 72 h (*n* = 3 biologically independent samples). Right: chemical structures and IC50 values of the TAME inhibitors.

Finally, we tested cell viability with an increasing concentration of SBFI-103, a member of the truxillic acid mono-ester (TAME) family of FABP5 inhibitors. These were previously shown to suppress the viability of human metastatic PC3 prostate cancer cells in vitro and in vivo [40,41]. As shown in Figure 3A, SBFI-103 effectively killed RCaP cells and four human cell lines, with IC_50_ values in the low micromolar range. Thus, our data show that murine tumor-derived RCaP cells, which are ADT and taxane resistant, are sensitive to TAME-based FABP5 inhibitors. A comparison of three SBFI analogs showed that SBFI-103 is most effective against RCaP cells (Figure 3B).

### 3.3. The FABP5-Inhibitor SBFI-103 Effectively Kills RcaP Tumor Cells In Vivo

To validate the FABP5-inhibitor sensitivity of RcaP cells and test the efficacy of the drug in vivo, we performed a series of trials of RcaP cell transplantation into mice. We used SBFI-103 because, besides showing the highest efficacy against RcaP cells in vitro, it previously demonstrated a good toxicity profile in vivo [41].

**Figure 4:**
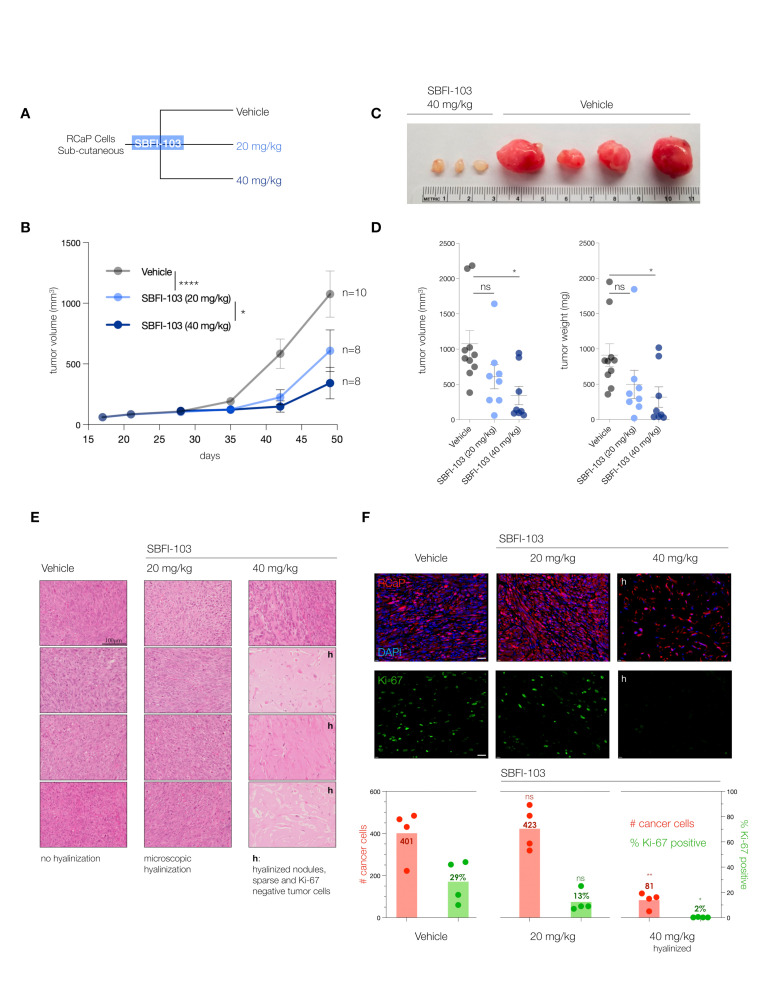
(**A**), Treatment scheme for in vivo testing of SBFI-103 at two doses in sub-cutaneously transplanted RCaP cells. (**B**), Tumor volumes and treatment effects in the three trial arms: vehicle, *n* = 10; SBFI-103 at 20 mg/kg, *n* = 8; SBFI-103 at 40 mg/kg, *n* = 8. Data are mean ± s.e.m. *p* values were calculated from a mixed-effects model (restricted maximum likelihood (REML)). The *p* values for comparison of vehicle with SBFI at 20 mg/kg (**** *p* < 0.0001) and SBFI at 20 mg/kg compared with 40 mg/kg (* *p* < 0.05) are indicated in the graph. The *p* value for comparison of vehicle with SBFI at 40 mg/kg is *** *p* < 0.001. (**C**), macroscopic analysis of select resected tumors from (**B**). See Supplementary Figure S3A for further examples. (**D**) Comparison of tumor volumes (left) and weights (right) among the three trial arms. Data are mean ± s.e.m. *p* values were calculated using one-way ANOVA with Dunnett’s post hoc test. Ns, not significant. * *p* < 0.05, **** *p* < 0.0001. (**E**) Histopathology analysis of lesions from (**B**) by H&E staining. “h” denotes high degree of hyalinization. Scale bar, 100 µm. (**F**), Top, Immunofluorescence analysis of histology slides from E showing RCaP cancer cells (red, tdTomatoFP), DAPI (blue), and anti-Ki-67 staining (green) in representative examples of the three trial arms. Scale bar 100 µm. Bottom, quantification of cancer cell number and percent Ki-67 positive cells per field for vehicle and 20 mg/kg trial arms and for hyalinized nodules of the 40 mg/kg trial arm (*n* = 4). *p* values were calculated using two-tailed Student’s *t*-test. * *p* < 0.05, ** *p* < 0.01.

The trials were performed in nude mice at two doses administered daily: 20 mg/kg SBFI-103 has previously demonstrated suppression of human PC3 cancer cells and was chosen as a starting point, while a 40 mg/kg drug was used to test for dose dependency (Figure 4A). After 30 days of treatment, we observed a significant 50% reduction in tumor volume at the lower drug dose, and treatment with the higher dose showed even further reduction of tumor volume by 75% on average when compared to the vehicle (Figure 4B). Postmortem analysis revealed even more substantial anti-tumor effects of SBFI-103. First, we noted that individual resected tumor volumes revealed that at the higher dose, 5 out of 8 lesions had dramatically regressed to below 200 mm^3^—an 80% reduction compared to vehicle (Figure 4C and Supplementary Figure S3A). Our analysis of tumor weights further showed that these 5 responder lesions had a 95% reduction in mass compared to the average vehicle treatment (Figure 4D). As shown in Figure 4E, histopathology analysis revealed that the vehicle-treated lesions presented with high-grade and poorly differentiated carcinoma and high mitotic figures (column 1, vehicle). In contrast, the high-dose-treated samples with low tumor volume showed only a few cancer cells, while the lesion was dominated by hyalinization, a hallmark indicator of cancer suppression (Column 3, 40 mg/kg, h). To more precisely define this anti-tumor effect, we performed a quantitative immunohistochemistry analysis. Since RCaP prostate tumor cells express tdTomato fluorescent protein, we used red fluorescence in combination with green-labeled Ki-67 proliferation marker and blue DAPI staining to quantify three parameters: number of cells per field, the number of cancer cells per field, and the number of Ki-67 positive cancer cells per field.

This analysis (see Figure 4F) showed that vehicle-treated lesions are composed mostly of cancer cells (TomatoFP-DAPI double positive), of which 30% on average are Ki-67 positive, indicative of sampling in the S-G2 phase of the cell cycle [42]. In stark contrast, the hyalinized small mass/volume lesions of 40 mg/kg treated animals showed only sparse numbers of tumor cells that were 98% negative for the proliferation marker Ki-67. This strongly suggested that the masses of these small lesions were dictated by the hyalinization process and not by tumor cells, because tumor cells were scarce in number and not proliferating.

**Figure 5:**
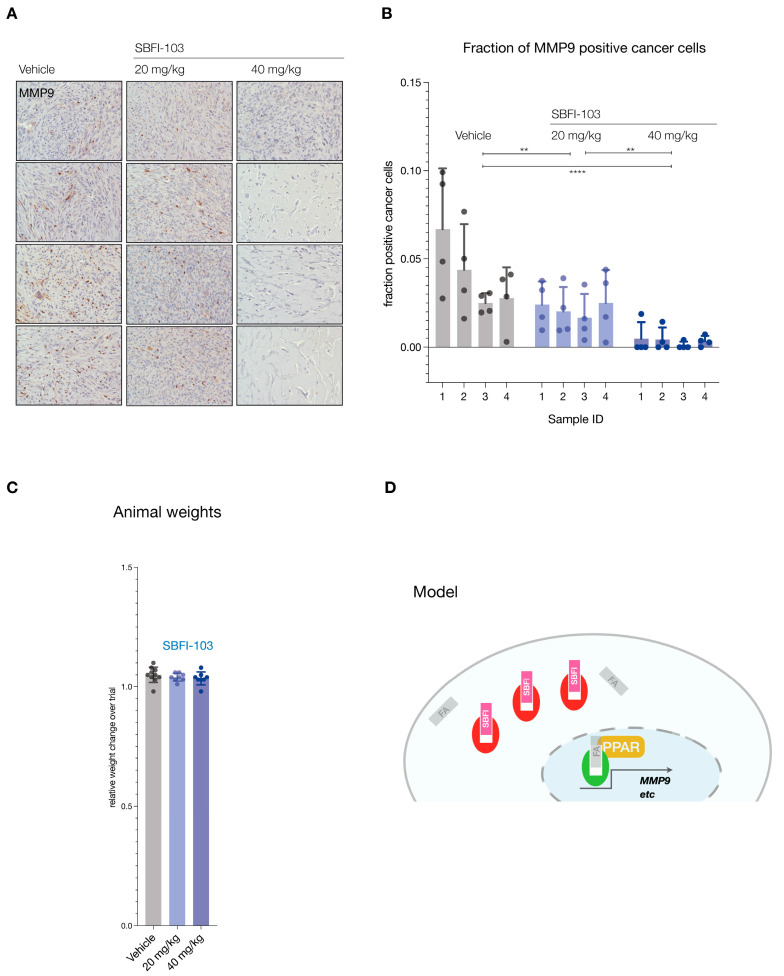
(**A**), Immunohistochemistry staining for MMP9 in tumors from 12 animals (vehicle, *n* = 4; SBFI-103 at 20 mg/kg, *n* = 4; SBFI-103 at 40 mg/kg, *n* = 4). Scale: panel width = 450 µm. (**B**), Quantification of MMP9-positive cancer cell fractions for the three trial arms. Individual data points show fraction of MMP9-positive cells per field divided by RFP-positive cells per field (see also Supplementary Figure S3B). Bars show mean ± s.d. *p* values were calculated using two-way ANOVA with Tukey’s post hoc test. ** *p* < 0.01, **** *p* < 0.0001. (**C**), fraction of change in total animal weights over trial period. (**D**), Model for inhibition of FABP5-mediated survival and its blockade with SBFI compounds. Green, active FABP5, red SBFI-inactivated FABP5. FA denotes a fatty acid ligand.

Finally, we tested functional inhibition by asking if SBFI-103 affected the FABP5 target MMP9 [43,44]. Analysis of the lesions demonstrated significant suppression and loss of MMP9 protein expression in the RFP-positive cancer cells (see Figure 5A for representative examples and Supplementary Figure S3B for all samples). Importantly, this suppression correlated with the SBFI-103 dose as quantified by IHC analysis of MMP9 expression per RFP-positive cell (Figure 5B). In contrast, we observed no treatment or dose-effects on animal weights (Figure 5C).

Based on the collective results from our in vivo experiments, we infer that at a well-tolerated dose of 40 mg/kg, SBFI-103 was able to eliminate the vast majority of prostate cancer cells, in spite of their resistance to in vitro ADT and taxane treatment.

## 4. Discussion

Significant progress with effective prostate cancer therapies has been made by moving from ligand regulation through androgen deprivation therapy (ADT) to direct blockade of the androgen receptor (AR). In spite of this, even innovative combination approaches are hardly ever able to achieve complete responses [45,46], and disease recurrence is the norm in the majority of patients. Therefore, it remains critically important to search for (1) novel targets, (2) effective drugs, and (3) novel model systems where new approaches against therapy-resistant PC can be rigorously tested. At the same time, loss of PTEN remains a major hallmark of aggressive PC, yet therapies directed at blockade of PI 3-Kinase have unfortunately shown limited success in PC. Even worse, setbacks in hematologic malignancies resulted in the shutdown of trials due to significant safety concerns in 2022 [47]. This has prompted the search for new vulnerabilities in cells driven by the loss of PTEN. Our work shows how validation of a new drug target combined with the development of castration/taxane-chemotherapy resistant prostate cancer cells can result in a much-needed platform for pre-clinical therapeutic research on lethal prostate cancer.

Similar to androgens, fatty acids (FAs) also control the activity of nuclear receptors. Critical for this signal transduction is the family of fatty acid-binding proteins (FABPs), which shield and solubilize FA lipid moieties and transfer them to activate nuclear receptors, foremost among them PPARγ [23,48] (see Cartoon, Figure 5D). The family of fatty acid-binding proteins comprises 10 paralogs in humans. This would suggest that there is much redundancy in function among the genes. Our data, however, strongly suggests that FABP5 stands out as a pivotal target in PC.

First, we demonstrate that FABP5 is among the most frequently amplified genes in human prostate cancer. This is by virtue of its genomic location near the MYC locus, a validated driver oncogene of PC on chromosome 8q [29,49,50]. Importantly, we find that when amplified at the DNA level, *FABP5* is also over-expressed at the RNA level, and this separates it from the other FABP genes, and especially the *FABP4*, *-9*, and *-12*, paralogs that are often co-amplified with FABP5 on chromosome 8. This suggests that molecular diagnostic tools that are routinely used in the clinic are already available for identification of those patients who could benefit from targeting FABP5 by profiling its genomic, transcriptomic, and, if needed, protein status.

Second, our research shows that the RapidCaP-derived RCaP cells can be used as proxies for ADT- and taxane-resistant human prostate cancer cells and that they are sensitive to FABP5 inhibitors in vitro and in vivo. This makes them suitable for future in vivo studies in mice that are fully immunocompetent, using orthotopic transplantation.

## 5. Conclusions

We show that in vivo targeting of androgen- and docetaxel-insensitive RCaP cells is possible using SBFI-103 at a well-tolerated dose of 40 mg/kg. Indeed, we find a strong elimination of tumor cells in 5 of 8 cases, suggesting that there is ample room for exploration of response mechanisms as well as a lack thereof. The actual process behind the therapeutic effect remains to be investigated. We note that both non-autonomous and cell-autonomous mechanisms could be at play. In this respect, our approach is compatible with the transplantation of mouse RCaP cells into the fully immunocompetent C57Bl6 mouse strain. Thus, future experiments using this system may yield insights that go beyond nude mouse transplantation of mouse or human PC cells because we can explore the drug’s effect and potential for synergy with a native tumor microenvironment. We propose that studying such effects may be of great value to future efforts at drug optimization.

## Data Availability

Data are contained within the article and supplementary materials and RNA sequencing data generated in this study have been deposited in the Gene Ex-pression Omnibus (http://www.ncbi.nlm.nih.gov/geo/) with the accession number GSM7951577.

## Supplementary Materials

The following supporting information can be downloaded at: https://www.mdpi.com/article/10.3390/cancers16010060/s1, Figure S1: Schematic for generation of RapidCaP derived cancer cell line - RCaP; Figure S2: Information on prostate cancer cell lines; Figure S3: Extended macropathology and immunohistology; Figure S4: Original Western Blot images.
