# Supplementary material for: FABP5 Inhibition against PTEN-Mutant Therapy Resistant Prostate Cancer
Source: Cancers (Basel). 2023 Dec 21;16(1):60. doi: 10.3390/cancers16010060 (PMC10871093; doi:10.3390/cancers16010060)
Supplement: Supplementary file 1 [file cancers-16-00060-s001.zip › cancers-2673421-supplementary.pdf]

Supplementary Figure S1

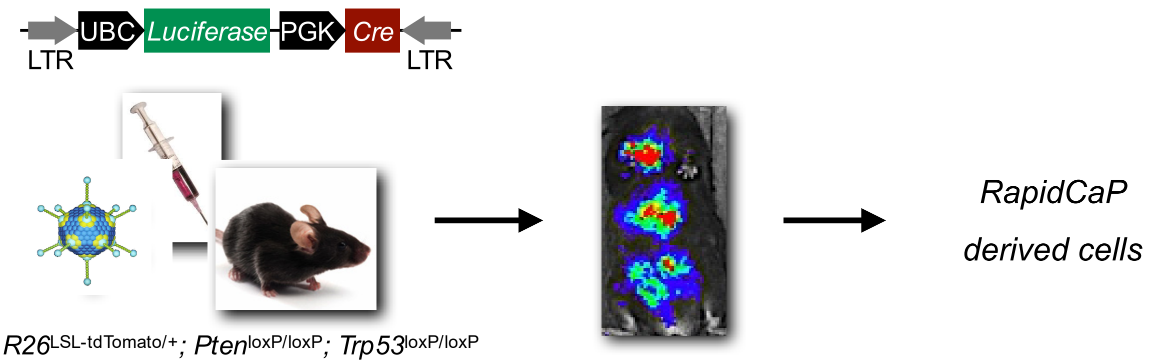

Supplementary Figure S2

A

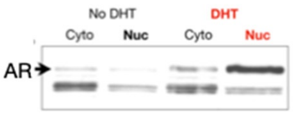

B

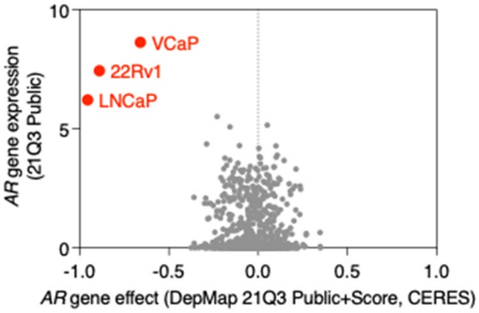

C

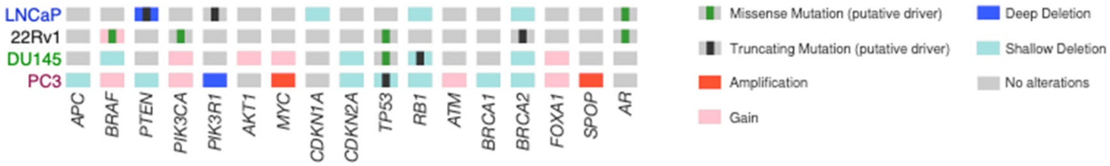

Supplementary Figure S3

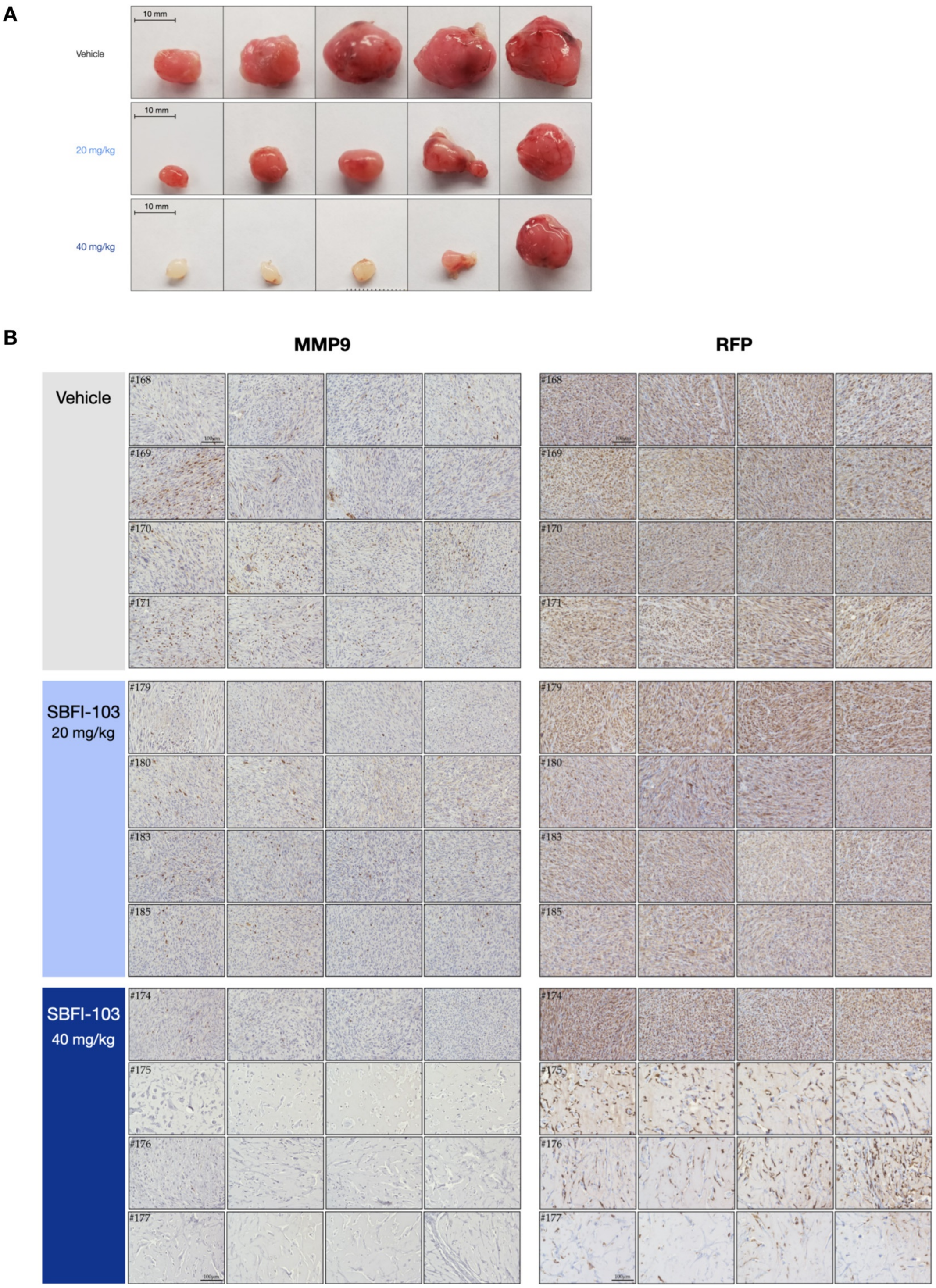

Figure S4: Original Western Blot images

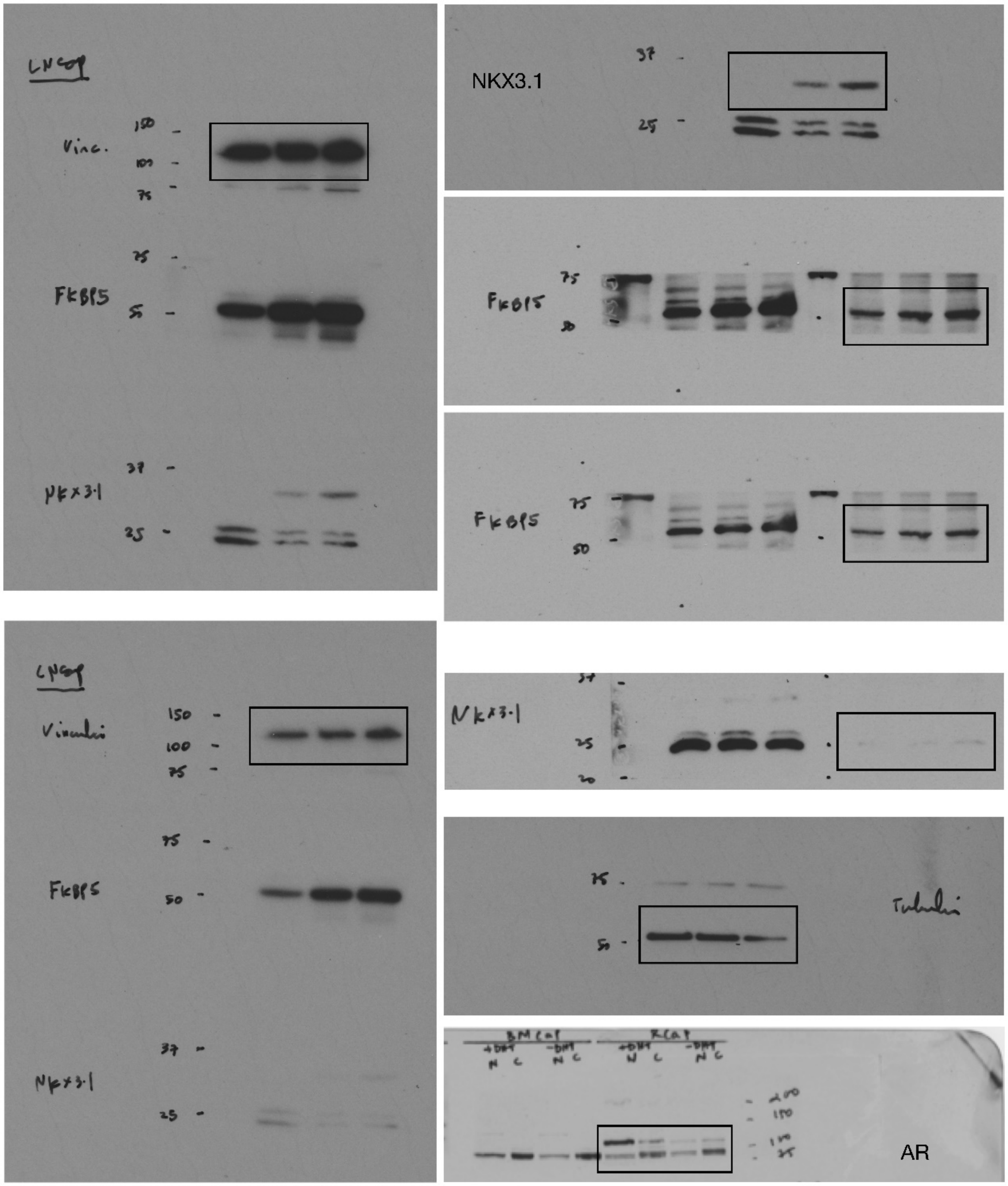

Figure S1. Schematic for generation of RapidCaP derived cancer cell line - RCaP.

Figure S2. : Information on prostate cancer cell lines

A, Western blot of androgen receptor in cytoplasmic and nuclear fractions from RCaP cells cultured in the absence and presence of DHT. B, *AR* genetic dependency and gene expression data in 1026 cancer cell lines from Broad's DepMap database (21Q3). AR gene-dependent prostate cancer cell lines are highlighted in red. C, Mutation frequency of prostate cancer associated tumor suppressor and oncogenes in the indicated prostate cancer cell lines curated at cBIOPortal.

Figure S3. Extended macropathology and immunohistology

A, Images of tumors resected from the trial animals. B, Immunohistochemistry staining for MMP9 (left) and RFP (right) in 4 tumor sections each from 12 animals. ID numbers are indicated (vehicle, n=4; SBFI-103 at 20 mg/kg, n=4; SBFI-103 at 40 mg/kg, n=4). Scale bar 100μm.

Figure S4. Original Western Blot images
